# Toxicity of locoregional radiotherapy in combination with bevacizumab in patients with non-metastatic breast cancer (TOLERAB): Final long-term evaluation

**DOI:** 10.1371/journal.pone.0221816

**Published:** 2019-08-30

**Authors:** Alice Clément-Zhao, Marie-Laure Tanguy, Paul Cottu, Brigitte De La Lande, Patrick Bontemps, Claire Lemanski, Pierre Baumann, Alexia Savignoni, Christelle Levy, Karine Peignaux, Agnès Reynaud-Bougnoux, Aline Gobillion, Youlia Kirova

**Affiliations:** 1 Radiotherapy Department, Institut Curie, Paris, France; 2 Biostatistics Department, Institut Curie, Paris, France; 3 Oncology Department, Institut Curie, Paris, France; 4 Radiotherapy Department, Institut Curie, Rene Huguenin Hospital, Saint Cloud, France; 5 Radiotherapy Department, CHU Jean Minjoz, Besançon, France; 6 Radiotherapy Department, Institut régional du Cancer de Montpellier, Montpellier, France; 7 Radiotherapy Department, Centre d’Oncologie de Gentilly, Nancy, France; 8 Radiotherapy Department, Centre François Baclesse, Caen, France; 9 Radiotherapy Department, Centre Georges-François Leclerc, Dijon, France; 10 Radiation Oncology Department, CHU Tours, Tours, France; MD Anderson Cancer Center, UNITED STATES

## Abstract

**Background and purpose:**

Few data are available concerning the safety of bevacizumab (B) in combination with locoregional radiation therapy (RT). The objective of this study was to evaluate the 5-year late toxicity of concurrent B and RT in non-metastatic breast cancer.

**Materials and methods:**

This multicentre prospective study included non-metastatic breast cancer patients enrolled in phase 3 clinical trials evaluating B with concurrent RT versus RT alone. All patients received neoadjuvant or adjuvant chemotherapy and normofractionated breast or chest wall RT, with or without regional lymph node RT. B was administered at an equivalent dose of 5 mg/kg once a week for 1 year. The safety profile was evaluated 1, 3 and 5 years after completion of radiotherapy.

**Results:**

A total of 64 patients were included between November 2007 and April 2010. Median follow-up was 60 months (12–73) and 5-year late toxicity data were available for 46 patients. The majority of tumours were triple-negative (68.8%), tumour size <2cm (41.3%) with negative nodal status (50.8%). Median total dose of B was 15,000mg and median duration was 11.2 months. No grade ≥3 toxicity was observed. Only 8 patients experienced grade 1–2 toxicities: n = 3 (6.5%) grade 1 lymphedema, n = 2 (4.3%) grade 1 pain, n = 1 (2.2%) grade 2 lymphedema, n = 1 (2.2%) grade 1 fibrosis. Five-year overall survival was 93.8%, disease-free survival was 89% and locoregional recurrence-free survival was 93.1%.

**Conclusion:**

Concurrent B and locoregional RT are associated with acceptable 5-year toxicity in patients with non-metastatic breast cancer. No grade ≥3 toxicity was observed.

## Introduction

Neoangiogenesis plays a central role in tumour growth and metastasis, with the vascular endothelial growth factor (VEGF)[1] acting as a key growth factor in breast tumours. Bevacizumab (Avastin^®^, Genentech Pharmaceuticals, San Francisco, CA) is a humanized monoclonal antibody targeting circulating VEGF.

Encouraging results have been reported with the combination of bevacizumab and chemotherapy in metastatic breast tumours[2].

Four clinical trials have been conducted to investigate the potential benefit of the combination of bevacizumab with standard neoadjuvant and/or adjuvant therapy in non-metastatic breast cancer.

- BEVERLY-1 trial (NCT00820547)[3] is a phase 2 study designed to determine the efficacy and safety of the combination of bevacizumab with neoadjuvant and adjuvant chemotherapy in patients with non-metastatic inflammatory breast cancer without HER2 overexpression
- BEVERLY-2 trial (NCT00717405)[4] is a phase 2 study designed to determine the efficacy and safety of the combination of bevacizumab with neoadjuvant and adjuvant chemotherapy and trastuzumab in patients with non-metastatic inflammatory breast cancer with HER2 overexpression
- BEATRICE trial (NCT00528567)[5,6] is a phase 3 study designed to determine the efficacy and safety of the combination of bevacizumab with adjuvant chemotherapy in patients with non-metastatic triple-negative breast cancer
- BETH trial (NCT00625898)[7] is a phase 3 study designed to determine the efficacy and safety of the combination of bevacizumab with adjuvant chemotherapy and trastuzumab in patients with non-metastatic breast cancer with HER2 overexpression

Despite the encouraging results of preliminary studies concerning the efficacy of bevacizumab in combination with chemotherapy in metastatic breast cancer[2], no clinical benefit was observed in these trials[3,5,7]. Only limited data are available concerning the safety of the combination of bevacizumab and locoregional radiotherapy, with heterogeneous results: toxicity has been described in phase I and II trials in lung cancer and pancreatic cancer[8–10], although this combination was well tolerated by patients with cervical cancer and pancreatic cancer in another phase II trial[11,12].

In the BEVERLY-1, BEVERLY-2, BEATRICE and BETH trials, bevacizumab was administered concurrently with locoregional radiotherapy.

To evaluate the safety of this combination, patients treated in France in these trials and randomized in the bevacizumab arm were enrolled in the TOLERAB (Toxicities of Locoregional Radiotherapy Associated with Bevacizumab in patients with non-metastatic breast cancer) study.

The final long-term (5 years) of toxicity results are presented here. Acute, one-year and three-year toxicity results have been previously published[13–15].

## Materials and methods

### Patients

TOLERAB, the French multicentre non-interventional single-arm observational cohort, included non-metastatic breast cancer patients treated by concurrent bevacizumab with local or locoregional radiotherapy. Patients received neoadjuvant or adjuvant chemotherapy in the BEVERLY 1, BEVERLY 2, BEATRICE or BETH trials [3–7]. Exclusion criteria were bilateral breast cancer, history of another cancer, medical conditions preventing the administration of bevacizumab, impossibility to attend long-term follow-up and inability to provide informed consent. All patients were informed about the study and they provide written consent. The study was conducted in accordance with the Hesinki declaration. According to French legislation, the study and the consent procedures were approved by two national independant committe: the « Comité Consultatif sur le Traitement de l’Information en matière de Recherche dans le domaine de la Santé » (CCTIRS) and the « Commission Nationale de l’Informatique et des Libertés » (CNIL).

### Treatment

All patients received neoadjuvant (in BEVERLY 1 and BEVERLY 2 trials) or adjuvant chemotherapy (in BEATRICE and BETH trials), in combination with trastuzumab in the case of histologically confirmed HER2-positive status.

Bevacizumab was administered with concurrent radiotherapy at an equivalent dose of 5 mg/kg every week intravenously (15 mg/kg every 3 weeks or 10 mg/kg every 2 weeks) for 1 year.

- In BEVERLY 1[3] and BEVERLY 2[4] trials, patients received bevacizumab 15 mg/kg every 3 weeks for eight injections in the neoadjuvant phase. Bevacizumab was stopped at least 4 weeks before surgery and restarted during or after radiotherapy, once the wound was healed entirely. Adjuvant bevacizumab (15 mg/kg every 3 weeks) was given for ten injections.
- In BETH trial[7], patients received locoregional adjuvant radiotherapy after completing adjuvant chemotherapy. Bevacizumab (15 mg/kg every 3 weeks) was started with adjuvant chemotherapy and continued it for a total duration of 1 year following first bevacizumab dose, without stop during radiotherapy.
- In BEATRICE trial[5,6], patients received locoregional adjuvant radiotherapy either before or after completing adjuvant chemotherapy, as per local guidelines. Patients started bevacizumab (15 mg/kg every 3 weeks or 10 mg/kg every 2 weeks) with adjuvant chemotherapy and continued it for a total duration of 1 year, without stop during radiotherapy.

Radiotherapy clinical target volumes included breast or chest wall, with or without regional lymph nodes. Volumes and fractionation were chosen in accordance with local practice and were already described in the 3-years and 1-year previous report [14–15].

### Endpoints

The primary endpoint was the safety of the concurrent combination of bevacizumab with radiotherapy in non-metastatic breast cancer. Acute toxicity was assessed in terms of radiation dermatitis and oesophagitis. Late toxicity was assessed at 12, 36 and 60 months according to the Common Terminology Criteria for Adverse Events version 3.0. Cases of pain, fibrosis, telangiectasia, lymphoedema, ulceration, myocardial infarction, pericarditis, dyspnoea, dysphagia and paresis were collected.

Secondary endpoints were the cosmetic results (according to the classification of Harris and al. [16]) and left ventricular ejection fraction (LVEF).

### Evaluation tools

The baseline evaluation comprised recording of medical history, WHO performance status, physical examination and a laboratory work-up. For the study, patients were evaluated by the radiation oncologist once a week during radiotherapy, 4 weeks after completion of radiotherapy and one, three and five years after treatment. Additionally, they also had a normal post-treatment follow-up at least every 6 months.

### Statistical analysis

Categorical variables were described by frequencies and percentages. Continuous variables were described by their means and/or medians with variances and/or ranges. Survival estimates were calculated with the Kaplan-Meier method. All analyses were performed with R version 3.4.2 software.

## Results

### Patient characteristics

From November 2007 to April 2010, 64 patients received concurrent bevacizumab concurrently with adjuvant radiotherapy for breast cancer. All patients received adjuvant bevacizumab, 24 patients received neoadjuvant and adjuvant bevacizumab (corresponding to patients included in BEVERLY 1 or BEVERLY 2 trials). Patients characteristics were already described in the 3-years previous report [15] and are shown in Table 1. Median age was 52.9 years (range: 23–68 years) and 60.9% of cases had cancer of the left breast. The most common histological type was invasive ductal carcinoma (89.1%) and the histological grade was III in 78.1% of cases. Hormonal receptors were positive in 18.8% of patients, HER2 receptor was overexpressed in 23.4% of cases and 68.8% of patients had triple-negative breast cancer. Median follow-up was 59.9 months (range: 12–73 months), the first quartile is 57.2 months, the third quartile is 62.3 months. Concerning the 18 patients without 5-year late toxicity data, nine of them died before 5 years and nine were lost to follow-up.

**Table 1:** Patient characteristics.

	N (n = 64)	*%*
**Menopause**	38	*59*.*4*
**Cardiovascular risk factor**		
Obesity (BMI>30)	13	*20*.*3*
Hypertension	10	*15*.*6*
Smoking	6	*9*.*4*
Diabetes	2	*3*.*1*
Deep arterial or venous thrombosis	1	*1*.*6*
Hyperlipideamia	3	*4*.*7*
**Age (years)** Median [range]	52.9 [23–68]	
**Side**		
Left breast	39	*60*.*9*
Right breast	25	*39*.*1*
**Histology**		
Invasive ductal	57	*89*.*1*
Invasive lobular	4	*6*.*2*
Others	3	*4*.*7*
**Clinical T stage**		
T0	2	*3*.*1*
T1	24	*37*.*5*
T2	14	*21*.*9*
T3	0	*0*
T4	23	*35*.*9*
Tx	0	*0*
Missing data	1	*1*.*6*
**Clinical N stage**		
N0	32	*50*
N1	24	*37*.*5*
N2	4	*6*.*2*
N3	3	*4*.*7*
Nx	0	*0*
Missing data	1	*1*.*6*
**UICC stage**		
Stage I	19	*29*.*7*
Stage IIA	17	*26*.*6*
Stage IIB	4	*6*.*2*
Stage IIIA	0	*0*
Stage IIIB	23	*35*.*9*
Missing data	1	*1*.*6*
**Histological grade**		
I	2	*3*.*1*
II	11	*17*.*2*
III	50	*78*.*1*
Anaplastic	1	*1*.*6*
**Hormone receptor status**		
HR+	12	*18*.*8*
HR-	52	*81*.*3*
**HER2 overexpression**	15	*23*.*4*
**Triple-negative breast cancer**	44	*68*.*8*

### Treatment characteristics

#### Surgery

Surgery consisted of total mastectomy for 44.4% of patients and breast conserving surgery for 55.6% of patients, with sentinel lymph node dissection in 32.8% of patients and axillary lymph node dissection in 82.8% of cases. The indication for axillary lymph node dissection was chosen in accordance with local practice.

Surgery was performed after neoadjuvant chemotherapy in 37.5% of patients.

#### Radiotherapy

Radiotherapy was given with conventional fractionation for 45 patients and 19 patients received hypofractionated radiotherapy (from 2.13Gy per fraction to 2.66Gy per fraction). Radiotherapy characteristics are shown in Table 2. Whole breast alone was treated in 56.3% of cases at a median dose of 50Gy and with a median tumour boost dose of 16Gy. Chest wall radiotherapy was treated in 43.7% of cases at a median dose of 49Gy. Lymph nodes radiotherapy was performed in 43 patients (67.2%) with supraclavicular and infraclavicular nodes, internal mammary nodes and axillary nodes in respectively 95.5%, 58.1% and 4.7% of the cases.

**Table 2:** Radiotherapy characteristics.

	N (n = 64)	*%*
**BREAST RADIOTHERAPY**	**36**	***56*.*3***
**Median dose (Gy), [range]**	50 [40–58]	
**Dose per fraction**		
≤ 2Gy	27	*75*
> 2Gy	9	*25*
**Irradiation technique**		
Dorsal decubitus	30	*83*.*3*
Lateral decubitus	5	*13*.*9*
Missing data	1	*2*.*8*
**Type of radiation**		
Photons	33	*92*
Cobalt	1	*3*
Missing data	2	*5*
**BOOST**	**36**	***56*.*3***
**Median dose (Gy), [range]**	16 [10–18]	
**Type of radiation**		
Photons	20	*55*.*5*
Electrons	10	*27*.*7*
Photons and electrons	5	*13*.*8*
Missing data	1	*3*
**CHEST WALL RADIOTHERAPY**	**28**	***43*.*7***
**Median dose (Gy), [range]**	49 [40–54]	
**Dose per fraction**		
≤ 2Gy	18	*64*.*3*
> 2Gy	10	*35*.*7*
**Type of radiation**		
Photons	14	*50*
Electrons	11	*39*.*3*
Photons and electrons	3	*10*.*7*
**LYMPH NODES RADIOTHERAPY**	**43**	***67*.*2***
**Supraclavicular nodes**	41	*95*.*4*
**Internal mammary nodes**	25	*58*.*1*
**Axillary nodes**	2	*4*.*7*

#### Systemic treatment

Median duration of bevacizumab was 11.7 months with median total dose of 15000mg (range 960–28080). Administration schedule was every three weeks for 91% of patients. Systemic treatment characteristics are shown in Table 3. Neoadjuvant chemotherapy was administered to 37.5% of patients. Adjuvant chemotherapy was administered to 63.5% of patients, comprising anthracycline and/or taxanes. Patients with HER2-overexpression received trastuzumab (23.4%) and hormonal therapy was administered to 14.1% of patients.

**Table 3:** Systemics treatments.

	N (n = 64)	*%*
**NEOADJUVANT CHEMOTHERAPY**	**24**	***37*.*5***
Sequential combination of taxanes and anthracyclines	24	
**ADJUVANT CHEMOTHERAPY**	**40**	***62*.*5***
Anthracycline-based chemotherapy only	3	
Taxane-based chemotherapy only	3	
Sequential combination of taxanes and anthracyclines	34	
**TRASTUZUMAB**	**15**	***23*.*4***
Median dose, mg [range]	7164 [3300–8370]	
Median duration, months [range]	12.17 [3.78–13.24]	
**HORMONTAL THERAPY**	**9**	***14*.*1***
Tamoxifen	5	
Aromatase inhibitor	4	
**BEVACIZUMAB**	**64**	***100***
Neoadjuvant and adjuvant	24	
Adjuvant only	40	
Neoadjuvant only	0	
Median dose, mg [range]	15000 [960–28080]	
Median duration, months [range]	11.7 [2.1–15.3]	

### 5-year toxicities

Forty-six patients (71.9%) were evaluated at 5 years. Only 8 patients reported late toxicity. The most common toxicities were grade 1 lymphoedema (n = 3, 6.5%) and grade 1 pain (n = 2, 4.3%). One patient experienced grade 1 fibrosis and one patient experienced grade 2 lymphoedema. No grade ≥3 toxicity was reported. These symptoms are in most cases related to the more aggressive surgery: total mastectomy and axillary lymph node dissection for the four patients with lymphoedema, breast conserving surgery and axillary lymph node dissection for the two patients with pain and for the patient with fibrosis.

No myocardial infarction, no pericarditis and no dyspnoea were reported. Details of the 5-year toxicities are shown in Table 4.

**Table 4:** Five-years toxicities.

	N (n = 64)	*%*
**5-YEARS TOXICITY EVALUATION**		
Yes	46	*71*.*9*
No	18	*28*.*1*
**5-YEARS TOXICITY**		
Yes	8	*17*.*4*
No	38	*82*.*6*
**Pain**		
Grade 1	2	*4*.*3*
Grade 2	0	*0*
Grade >2	0	*0*
**Fibrosis**		
Grade 1	1	*2*.*2*
Grade 2	0	*0*
Grade >2	0	*0*
**Telangiectasia**		
Grade 1	0	*0*
Grade 2	0	*0*
Grade >2	0	*0*
**Lymphoedema**		
Grade 1	3	*6*.*5*
Grade 2	1	*2*.*2*
Grade >2	0	*0*
**Ulceration**		
Grade 1	0	*0*
Grade 2	0	*0*
Grade >2	0	*0*
**Myocardial infarction**		
Grade 1	0	*0*
Grade 2	0	*0*
Grade >2	0	*0*
**Pericarditis**		
Grade 1	0	*0*
Grade 2	0	*0*
Grade >2	0	*0*
**Dyspnea**		
Grade 1	0	*0*
Grade 2	0	*0*
Grade >2	0	*0*
**Dysphagia**		
Grade 1	0	*0*
Grade 2	0	*0*
Grade >2	0	*0*
**Paresis**		
Grade 1	0	*0*
Grade 2	0	*0*
Grade >2	0	*0*
**Other**	1	*2*.*2*

LVEF evaluation at 5 years was available for 24 patients (37.5%) and only one patient had an LVEF value less than 50%.

Among the 35 patients treated by breast-conserving surgery, 12 were evaluated for cosmetic results and only one patient reported a cosmetic modification (grade 1).

### Patient outcomes

Five-year overall survival, progression-free survival and distant progression-free survival were 90.4% (95% Confidence Interval: 83–98%), 90.4% (83–98%) and 90% (85–95%), respectively.

## Discussion

This French observational study, based on prospective data from the main trials evaluating adjuvant or neoadjuvant bevacizumab therapy for non-metastatic breast cancer, describes acceptable 5-year toxicities of the combination of bevacizumab and locoregional radiotherapy with no grade ≥3 toxicity.

This is the largest published study with the longest follow-up reporting the late toxicity of the combination of bevacizumab and locoregional radiotherapy for non-metastatic breast cancer. At 5 years, only 17.4% of patients evaluated (n = 8/46) reported toxicity, all grade 1–2 and the most common toxicities were lymphoedema and pain. More toxicities were reported at 3 years in the same cohort,[15],: grade 1 (mainly lymphoedema and pain) for 39.5% of patients evaluated (n = 18/43) and only one patient experienced grade 3 lymphoedema. A favourable outcome in terms of acute toxicity has been previously reported [14,17].

A limitation of this study is the high rate of missing data for LVEF and it is consistent with a previously French study[18]. One explanation could be the small proportion of patients treated by trastuzumab and therefore not requiring regular cardiological screening. However, the lack of LVEF evaluation may be compensated by the report of myocardial infarction, pericarditis and dyspnoea. Five-years toxicites were available for 46 patients (71.9% of patients) and none of them reported these cardio-pulmonary toxicities.

Although there is a rationale for use of concurrent bevacizumab and radiotherapy (inhibition of VEGF leads to transient normalization of tumour oxygenation, minimizing hypoxia and consequently inducing decreased radiation sensitivity [19]), few data are available concerning the late toxicity of this combination, with heterogeneous results. A high rate of grade 2–3 toxicities was observed for high-risk prostate cancer [20], which may have been worsened by bevacizumab, with a median follow-up of 34 months. For non-small cell lung cancer, major oesophageal toxicities were reported in the first year [8,21] and late toxicity was therefore not investigated. In a phase 3 trial in glioblastoma [22], more grade ≥3 toxicities were observed with bevacizumab than without bevacizumab, with a median follow-up of 12.3 months. In contrast, a phase 2 trial in advanced nasopharyngeal carcinoma [23] reported 20% of grade 1–2 treatment-related haemorrhage and no grade 4–5 late toxicity, with a median follow-up of 30 months, and concluded that this treatment is feasible. A phase 2 trial in advanced cervical carcinoma [11] also indicated the feasibility of this treatment with no treatment-related serious adverse events, with a median follow-up of 12.4 months.

To our knowledge, this is the first study to report the favourable 5-year safety profile of the combination of bevacizumab and radiation therapy.

Recent phase II and III trials [3,5,7] have failed to demonstrate any clinical benefit of adding bevacizumab to adjuvant chemotherapy in non-,metastatic breast cancer but longer follow-up and correlative studies to identify patients who might benefit from bevacizumab are needed.

In the metastatic setting, initial therapy with paclitaxel plus bevacizumab provided significant improvement of progression-free survival [2,24], but no study has shown any OS benefit [25,26]. Moreover, numerous retrospective studies have demonstrate the benefit of treating the primary tumour in metastatic breast cancer patients, particularly by means of radiation therapy [27].

In daily clinical practice, radiation oncologist are often in the position to evaluate whether it is safe for the patient to add radiotherapy to the ongoing treatment with bevacizumab. Our study could help to this decision.

## Conclusion

In conclusion, concurrent bevacizumab and locoregional radiation therapy for breast cancer did not induce any severe late toxicity at five years.
